# Petunia as model for elucidating adventitious root formation and mycorrhizal symbiosis: at the nexus of physiology, genetics, microbiology and horticulture

**DOI:** 10.1111/ppl.12762

**Published:** 2018-07-31

**Authors:** Uwe Druege, Philipp Franken

**Affiliations:** ^1^ Leibniz Institute of Vegetable and Ornamental Crops Erfurt 99090 Germany

## Abstract

Adventitious root formation in cuttings and establishment of arbuscular mycorrhizal symbiosis reflect the enormous plasticity of plants and are key factors in the efficient and sustainable clonal propagation and production of ornamental crops. Based on the high importance of *Petunia hybrida* for the European and US annual bedding plant markets and its suitability as a model for basic plant sciences, petunia has been established as an experimental system for elucidating the molecular and physiological processes underlying adventitious root formation and mycorrhizal symbiosis. In the present review, we introduce the tools of the *Petunia* model system. Then, we discuss findings regarding the hormonal and metabolic control of adventitious rooting in the context of diverse environmental factors as well as findings on the function of arbuscular mycorrhiza related to nutrient uptake and resistance to root pathogens. Considering the recent publication of the genomes of the parental species of *P. hybrida* and other tools available in the petunia scientific community, we will outline the quality of petunia as a model for future system‐oriented analysis of root development and function in the context of environmental and genetic control, which are at the heart of modern horticulture.

**Abbreviations**
ACO: 1‐aminocyclopropane‐1‐carboxylic acid (ACC) oxidase
ACS: ACC synthase
AM: arbuscular mycorrhiza
AP2: APETALA2
AP‐UL: apoplastic unloading
AR: adventitious root
ARF: auxin response factor
Aux/IAA: auxin repressor
CACI: citrate cycle intermediates
D27: carotene isomerase D27
DAD2 (decreased apical dominance 2): α,ß hydrolase involved in strigolactone perception
DLI: daily light integral
GH3: Gretchen Hagen 3
Glc6PDH: glucose‐6‐phosphate dehydrogenase
GWAS: genome‐wide association study
HXK: hexokinase
IAA: indole‐3‐acetic acid
INVcw: cell wall invertase
MIR: mycorrhiza‐induced resistance
PAT: polar auxin transport
PFK: phosphofructokinase
P_n_: net photosynthesis
PPFD: photosynthetic photon flux density
SAUR: small auxin‐up RNAs
SNRK1: sucrose non‐fermenting 1‐related protein kinase 1
SYM‐UL: symplastic unloading
TIR1: transport inhibitor response 1
TPP: trehalose‐6‐phosphate phosphatase
TPS: trehalose‐6‐phosphate synthase
VIGS: virus‐induced gene silencing

## Introduction

The genetic and environmental control of root development and function are among the fundamental topics in plant science. Understanding these topics provides a high potential for improving the breeding of new cultivars in agriculture, horticulture and forestry and for their economic and ecologically sustainable production. *Arabidopsis thaliana* (Arabidopsis) has been intensively developed as the model of choice for fundamental plant science around the world since the 1980s, and its exploitation has provided a trove of knowledge about the genetic, molecular and physiological control of plant development. However, because of the habitus and lifestyle of Arabidopsis, immediate translation of Arabidopsis‐derived knowledge to other, mostly more complex growing crop plants, is often difficult.

In horticulture, the breeding and production of ornamentals constitutes an economically important sector involving the most sophisticated technologies developed and employed by specialists in plant genetics, physiology, nutrition and health. Great challenges for future ornamental crop production include the efficient use of resources such as nutrients, water and energy and minimum pollution of the environment by chemicals. Many important ornamental crops are vegetatively propagated via adventitious root (AR) formation from shoot tip cuttings, a process that provides several billion rooted ornamental plants to the European market each year. However, the lack of understanding regarding the endogenous and environmental control of AR formation in cuttings impairs the smart breeding of cultivars for efficient propagation and frequently causes losses in young plant production. Most ornamental crop species can establish a symbiotic interaction with arbuscular mycorrhiza (AM) fungi, which can improve nutrient acquisition and stress tolerance in their hosts. Understanding the regulation and function of these symbioses is of central importance for providing efficient inocula and environmentally stable application protocols for AM as one element in sustainable horticultural production.

The garden petunia is vegetatively propagated from shoot tip cuttings and is able to form AM symbioses. It ranks among the top annual bedding plants in terms of wholesale value worldwide, particularly in Europe [6% market share; BMEL (2014)] and the United States [11% market share; Warner and Walworth (2010)]. *Petunia* was originally described by Jussieu (1803). Commercial petunia cultivars as well as the standard laboratory lines, which will be introduced below, have a hybrid origin. Since the 1950s, when geneticists began trying to predict new flower color classes of *Petunia hybrida*, research proliferated, powered by the development of biochemical and molecular methods, and now covers all aspects of plant science (Gerats and Vandenbussche 2005, Vandenbussche et al. 2016). There is great exchange of knowledge and resources within the *Petunia* research community, which is organized via the community‐driven PetuniaPlatform (http://flower.ens‐lyon.fr/PetuniaPlatform/PetuniaPlatform.html). Because of its importance for horticulture and its ability to serve as a model, the *Petunia* system has been increasingly applied and elaborated by our group and others to analyse the molecular and physiological regulation and function of AR formation and AM symbiosis. In the present review, we will first introduce the tools available for research on *Petunia*. Thereafter, we will provide an overview of how these tools have contributed to the current understanding of AR formation and AM symbiosis. Finally, we will discuss the outlook of future research.

### *Petunia hybrida* as model: the tool box

Fundamental biological sciences are based on the use of model organisms. In the field of plant research, *Arabidopsis thaliana* was the first model species (Kaul et al. 2000), mainly as a result of its relatively small genome, because large‐scale sequencing projects were initially very time‐consuming and costly. Arabidopsis regenerates ARs from excised leaves, intact hypocotyls of etiolated seedlings and de‐rooted hypocotyls of seedlings as well as thin cell layers and stem segments excised from the inflorescence stem (Correa et al. 2012, Gutierrez et al. 2012, Della Rovere et al. 2013, Verstraeten et al. 2013). However, the typical structure of a leafy shoot tip cutting, which is used for the clonal propagation of many ornamental crops, is undeveloped. Arabidopsis also does not form mycorrhizal symbioses. Since the beginning of the current decade, genome size has lost its dominant influence on the choice of models because of the development of new sequencing techniques, referred to collectively as next‐generation sequencing methods. Genome sequencing is now a relatively fast and low‐cost procedure; therefore, other criteria have become more important for determining the plants used in the investigation of research questions.

**Figure 1:**
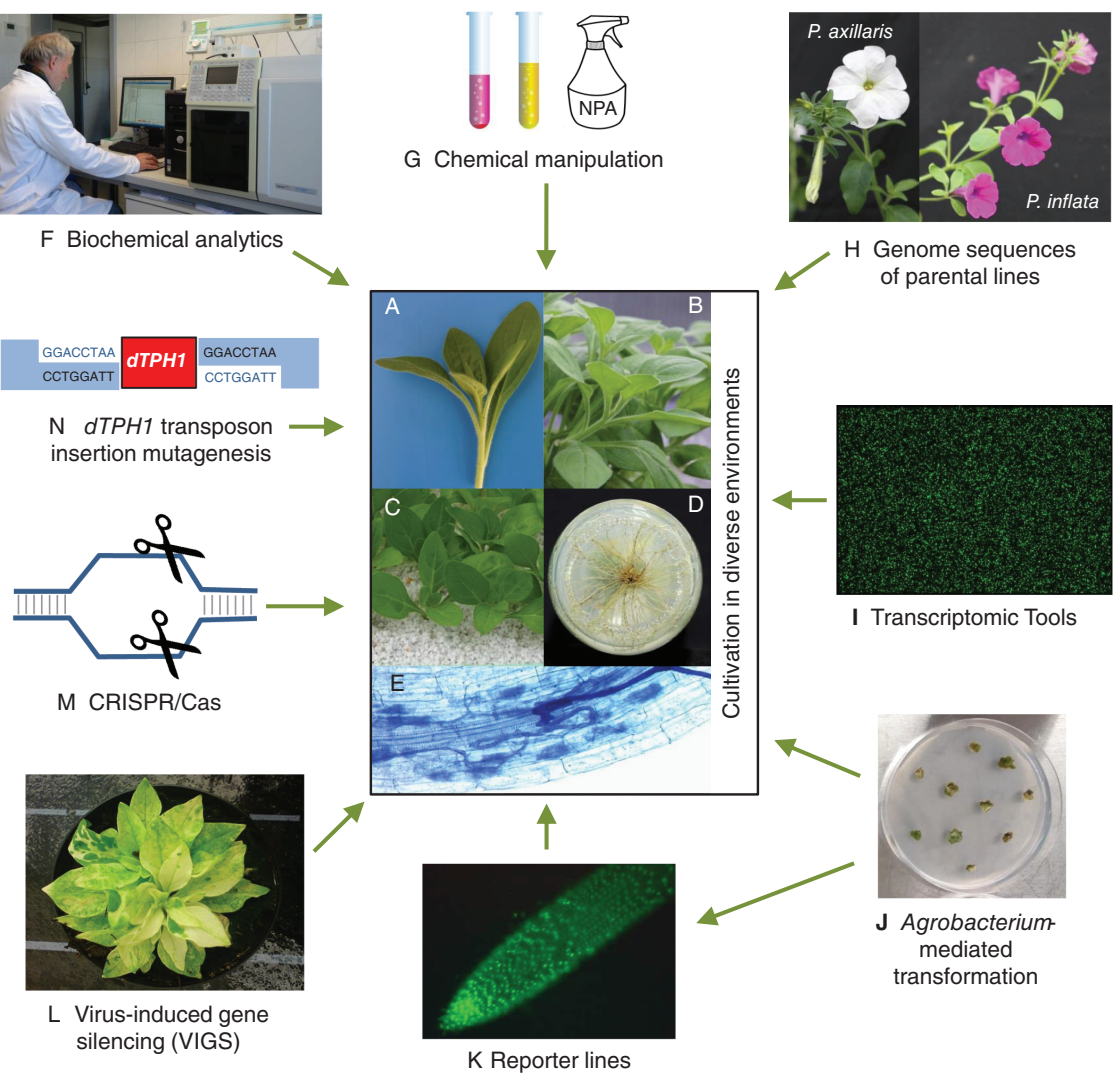
Selected *Petunia* genotypes and growth characteristics and the *Petunia* tool box. Shoot tip cuttings of *Petunia hybrida* ‘Mitchell’ (A) freshly excised from (B) the stock plant, (C) planted and cultivated in perlite and (D) with ARs produced when cultivated in agar medium in vitro (view from the bottom). (E) Structures of *Funneliformis mosseae* in the roots of *P. hybrida* ‘Mitchell’. (F) GC–MS/MS analysis of plant hormones. (G) Chemical manipulation, example of PAT inhibition by NPA. (H) Flowering shoots of sequenced *P. axillaris* and *P. inflata*. (I) *Petunia* microarray. (J) *Agrobacterium*‐mediated leaf disc transformation of *P. hybrida* ‘Mitchell’. (K) green‐labeled YFP‐fluorescence of the auxin‐response in ARs, *DR5::3xVenus‐6 in P. hybrida* ‘Mitchell.’ (L) virus‐induced silencing of the endogenous *phytoene desaturase* (*PDS*) gene, causing photo‐bleaching in *P. hybrida* ‘Fantasy Blue’, photo: A. Langhans, Prof. Dr. H. Mibus. Schematic illustration of (M) CRISPR/Cas and (N) *dTPH1* insertion mutagenesis.

Cultivation of *P. hybrida* can be conducted under a broad range of temperature and humidity conditions and does not require much space. In ideal conditions, up to four generations can be produced in 1 year. Moreover, masses of genetically identical material can be obtained by collecting shoot tip cuttings (Fig. 1A) from stock plants (Fig. 1B). In greenhouses or climate chambers (Fig. 1C) or under in vitro cultivation (Fig. 1D), such cuttings develop roots in approximately 2 weeks. Rooted cuttings can be directly used for various investigations, such as for studying AM symbiosis (Fig. 1E). During the last decade, a large set of analytical methods has been successfully applied to petunia, including tandem mass spectrometry of plant hormones (Fig. 1F). As described by Vandenbussche et al. (2016), the choice of the appropriate cultivar and the application of particular cultivation techniques, such as the removal of axillary shoots, allow the cultivation of large numbers of individual plants in a small area, and these plants continuously produce flowers over more than 1 year. These features of *P. hybrida* are ideal prerequisites for large screening experiments.

All modern *P. hybrida* cultivars and lines used for research purposes, such as *P. hybrida* ‘Mitchell’ (Fig. 1A–E), represent crosses originally derived from different wild species (Segatto et al. 2014). Crossing barriers do not exist between these species, and such crosses are therefore easy to conduct. Hence, populations of recombinant inbred lines (RILs) have been generated, and corresponding maps have been established, which have been used for the identification of quantitative trait loci (QTLs) for characters such as flower morphology (Galliot et al. 2006) and plant quality (Vallejo et al. 2015). Markers of these QTLs can be subsequently used in breeding programs to target the generation of new cultivars with desired traits in a relatively short time (Guo et al. 2017). Moreover, such loci are very likely to harbor so‐called candidate genes that may be responsible for the expression of these traits. In 2016, the genomes of *Petunia axillaris* and *Petunia inflata* (Fig. 1H), the wild parents of *P. hybrida*, were published (Bombarely et al. 2016). It is therefore possible to identify the sequences of putative candidate genes in QTLs that play a role in the trait or process under investigation.

In addition to the existence of wild species, an enormous variety of different cultivars exist that show wide‐ranging variability in their morphological parameters. These cultivars can further serve as a source for the identification of candidate genes through correlating sequence variations with phenotypic differences based on a genome‐wide association study (GWAS). Genes that putatively play a role in particular processes can be identified by their differential expression patterns. This was first carried out in a large‐scale experiment through hybridization arrays (Fig. 1I), based on all RNA sequences that were available before the sequencing of the petunia genomes (Breuillin et al. 2010). Such expression analyses are now carried out via deep sequencing of RNA extracts, with subsequent quantification of the different sequence types obtained and mapping to the corresponding genomes (Rich et al. 2017).

When putative candidate genes are identified through the described approaches, their role has to be further confirmed, which can be achieved via overexpression or downregulation using RNAi constructs of those genes and in‐depth analyses of the resulting transgenic plants. Prerequisite for such analyses is a suitable transformation system for the generation of stably transformed plants, which has been available for petunia since the 1980s (Horsch et al. 1985). The generation of transgenic petunia plants with orange‐colored flowers (Meyer et al. 1987), the knowledge gained from instabilities in color phenotypes (e.g. Linn et al. 1990), the political discussions about the first field experiments (Jasanoff 2005) and the recent re‐appearance of these phenotypes among commercial cultivars (Bashandy and Teeri 2017) are components of one of the most interesting stories in plant science history. The well‐established *Agrobacterium*‐mediated leaf disc transformation method (Fig. 1J) can also be used to localize the activity of target genes with the help of promoter‐reporter constructs (Fig. 1K). In addition to generation of stable transformants, numerous techniques can be applied to petunia for transient assays in a first screening approach, such as infiltration of leaves or flowers (e.g. Verweij et al. 2008) or virus‐induced gene silencing (VIGS) (Spitzer et al. 2007) (Fig. 1L).

The transfer of overexpression or RNAi constructs into plants only generates differences in the expression levels of candidate genes. However, it is often necessary to create a knock‐out mutation to produce a phenotype that is sufficiently strong to be observed. For this purpose, the methods of choice include insertion mutagenesis and newer options such as CRISPR/Cas technology (Fig. 1M), which has recently been applied in *P. hybrida* ‘Mitchell’ (Zhang et al. 2016). While the former approach usually leads to random insertions because of the lack of homologous recombination in plants, the latter can be targeted to particular genes of interest. In both cases, genetic engineering approaches are necessary, accompanied by rules and regulations making work with such plants more or less inconvenient. It is not clear what the result of the current discussion in Europe concerning CRISPR/Cas will be, but it will probably take years until this issue is clarified. However, insertion of sequences into genes also naturally occurs through transposable elements. Petunia harbors such a natural insertion mutagenesis system. The transposable element *dTPH1* inserts into genomic regions, and its excision leaves behind a target site duplication of eight nucleotides (Fig. 1N) (Gerats et al. 1990, 2013). Based on the *dTPH1* transposable element system in the *P. hybrida* line W138, a progeny population was generated (Vandenbussche et al. 2008). Application of the three‐dimensional polymerase chain reaction (PCR) genotyping approach to this population has contributed to the identification of insertion mutations in genes of interest (Vandenbussche et al. 2008, 2016). The corresponding mutant lines can subsequently be employed in functional studies, using the corresponding revertants as controls.

In summary, the genus *Petunia* exhibits high genetic variability, including crosses between different petunia species with corresponding maps, numerous cultivars with particular phenotypes and lines with insertion mutations. Second, the amount of available sequence information, including genome and RNA sequences annotated to a high degree, allows direct access to the molecular basis of this variability. Third, techniques for transient and stable transformation enable the functional analysis of genes for temporal and spatial analyses of gene activities and the localization of gene products. The skilled application of a combination of these tools allows the identification of candidate genes and their functional analysis in a straightforward manner.

## Research on adventitious root formation in petunia

Adventitious root formation in shoot tip cuttings, consisting of a leafy stem with the stem base as the rooting zone, the terminal shoot apex, and at least one fully developed leaf (Fig. 1A), is the crucial physiological process utilized for vegetative propagation of many ornamental plant species. In response to excision, a new developmental programme is initiated in specific plant cells, mainly in the cambium or vascular tissues. The initial induction phase, devoid of any cellular changes, is followed by the formation phase, which can be further subdivided into the initiation phase ending with the formation of dome‐shaped root primordia and the final expression phase, when the complete root body connected to the vascular cylinder is formed and emerges from the stem (da Costa et al. 2013).

AR formation is regulated by plant hormones. Hormonal regulation of AR formation in *P. hybrida* initially received research attention in the 1990s and early 2000s. The responses of petunia cuttings to the application of ethylene (ET; Dimasi‐Theriou et al. 1993) and the transgenic modulation of ET reception or down‐stream signaling (Clark et al. 1999, Shibuya et al. 2004) or cytokinin (CK) biosynthesis (Clark et al. 2004) revealed a positive role of ET activity and an inhibitory role of CK in AR formation. In 2006, a cooperative project was launched in Germany to develop *P. hybrida* as a model system for analyzing AR formation and AM symbiosis (see also next chapter). In the context of AR formation, research has focussed on molecular, hormonal and metabolic regulation in the stem base and the functional equilibrium of whole cuttings in response to environmental factors. The outcomes and related findings of other groups are summarized below.

### Analysis of the stem base

Ahkami et al. (2009) monitored the stem base of *P. hybrida* ‘Mitchell’ cuttings under standard rooting conditions (Fig. 1C) and discovered three metabolic stages: (1) the sink establishment phase, characterized by the induction and activation of cell wall invertase (INVcw), responsible for apoplastic unloading (AP‐UL) of sucrose; (2) the recovery phase, when the initially depleted sugars and amino acids recover; and (3) the maintenance phase, characterized by symplastic unloading of sucrose (SYM‐UL). A subsequent study conducted under the same conditions using a petunia microarray (Fig. 1I) confirmed the metabolic concept at the transcriptional level and pointed towards a possible role of peroxisomal ß‐oxidation, trehalose metabolism and sugar sensing during the early steps of AR formation (Ahkami et al. 2014). Furthermore, the induction of several mineral nutrient‐related genes and corresponding upregulation of ribonucleotide reductase (RNR), especially during the recovery and maintenance phases, indicated that nutrient acquisition and DNA synthesis are important processes in the rooting zone.

Continuous application of combined fertilizer to the stem base of two commercial petunia cultivars promoted AR formation, whereas leaf fertigation did not have such an effect (Santos et al. 2009). Recently, Hilo et al. (2017) shed light on the local functions of iron (Fe) and ammonium (NH_4_) in AR formation in the ‘Mitchell’ cultivar. They found striking local stimulation of AR formation by Fe and a promoting effect of NH_4._ The optimal application period for these nutrients corresponded to the early divisions of meristematic cells in the stem base and coincided with increased transcript levels of mitotic cyclins. Based on the analogous responses to NH_4_ and Fe and the finding that NH_4_ stimulated Fe accumulation in the stem base, Hilo et al. (2017) suggested that NH_4_ may act via enhancement of Fe acquisition based on the physiological acidification of the apoplast, which is a well‐established mechanism in the rhizosphere (Marschner 2012).

Ahkami et al. (2013) combined GC–MS/MS analysis of plant hormones and monitoring of primary metabolism and anatomy in the stem base of the ‘Mitchell’ cultivar with chemical manipulation to study the relationship between polar auxin transport (PAT), the temporal distribution of indole‐3‐acetic acid (IAA) and the function of auxin during AR formation in cuttings. Spraying the cuttings with the PAT blocker naphthyphtalamic acid (NPA) immediately after excision not only eliminated the 24 h peak of IAA and strongly inhibited AR meristemoid formation, but further reduced the early activity of vacuolar invertase and INVcw. Another microarray analysis performed under standard rooting conditions revealed complex regulation of genes controlling plant hormone homeostasis, signaling and downstream processes (Druege et al. 2014). In particular, transcriptomic fine‐tuning of the genes controlling PAT and other components affecting local auxin homeostasis was observed. Beyond the observed down‐regulation of genes encoding components of the transport inhibitor response 1 (TIR1) auxin receptor complex and many auxin response factors (ARFs), the transcript levels of genes encoding specific ARFs, many auxin‐repressing Aux/IAA proteins and small auxin‐up RNA (SAUR) proteins showed phase‐specific patterns of up‐ or downregulation, which may indicate important functions in the phase control of AR formation. Druege et al. (2014) postulated that the disruption of the basipetal auxin efflux through cutting excision is a major factor contributing to early auxin accumulation, which in turn triggers canalization of auxin to AR founder cells, where it initiates cellular reprogramming involving crosstalk with other hormones. Accordingly, phase‐specific regulation of genes coding for auxin‐responsive cyclins, for the ethylene response element binding protein APETALA2 (AP2) and for GRAS transcription factors (major players in auxin and gibberellin signaling), was recorded in the stem base (Ahkami et al. 2009, 2014, Bombarely et al. 2016).

Highlighting the contribution of ET, many genes involved in ET biosynthesis and signaling was found to be strongly upregulated throughout the period of AR formation, some of which are also induced in leaves through wounding (Druege et al. 2014). The rooting phenotypes observed in response to inhibitors of ET biosynthesis or perception as well as in response to the ET‐precursor 1‐aminocyclopropane‐1‐carboxylic acid (ACC) confirmed the positive role of ET in AR formation but indicated an inhibitory role of ET in AR elongation. Jasmonic acid (JA) is another wound‐responsive hormone that has recently been considered in relation to AR formation. After high early accumulation of JA was detected in the stem base of the ‘Mitchell’ cultivar (Ahkami et al. 2009), genetic transformation of this cultivar revealed that JA production is required for maximum AR formation. Marked reduction of petunia allene cyclase oxide synthase transcript levels and activity not only reduced the level of wound‐induced JA and its bioactive conjugate in cuttings, but also further reduced the number of root primordia and the final number of ARs (Lischweski et al. 2015).

Strigolactones (SL) are a class of carotenoid‐derived plant hormones that, after being discovered as stimulants of the germination of parasitic weeds, were shown to have an important function in AM symbiosis (see next chapter) and to control plant development in different ways (Al‐Babili and Bouwmeester 2015). The response of AR formation in cuttings of *Solanum lycopersicum* and *Pisum sativum* to genetic and chemical modifications of SL biosynthesis, levels and responses revealed a negative role of SLs in AR primordium development and in final rooting (Kohlen et al. 2012, Rasmussen et al. 2012). Interestingly, the expression of important genes controlling SL biosynthesis and perception was found to be reduced in the stem base of petunia soon after cutting excision (Bombarely et al. 2016). This finding indicates that, in addition to the postulated dilution resulting from the removal of the root system (Steffens and Rasmussen 2016), local down‐regulation of SL biosynthesis and signaling may also contribute to AR formation in the stem base of cuttings.

### Analysis at whole‐cutting level

The supplementation of the carbohydrate reserves present in cuttings at the time of planting through net photosynthesis during cutting cultivation provides an important carbon source. Cuttings of the ‘Mitchell’ cultivar show a high net photosynthetic rate (P_n_) from the first day after excision onwards and positive short‐term responses to increases in the photosynthetic photon flux density (PPFD) and CO_2_ concentrations, while dark respiration is increased under higher air temperatures (Klopotek et al. 2012). The acclimation of cuttings to a lower PFFD for 1 week significantly reduced the maximum P_n_ value and the light compensation point of fully developed source leaves; similar responses were observed in intact shoots still attached to the stock plant. Accordingly, an increase of the daily light integral (DLI) enhanced maximum gross photosynthesis, the light compensation point of photosynthesis and starch levels in cuttings of a commercial petunia cultivar, which corresponded to increased dry matter production of leaves, stem and ARs (Currey and Lopez 2015). In Arabidopsis, the perception of the light spectrum is strongly linked to the plant hormone pathways in which PHYTOCHROME‐INTERACTING FACTORS affect ARFs, Aux/IAA proteins and AP2 transcription factors (de Lucas and Prat 2014). Interestingly, enhancing the blue and red fractions during supplemental LED lighting of petunia cuttings was shown to increase the leaf dry mass, root dry mass, percentage of root dry mass within the total dry mass and the root to shoot ratio (Currey and Lopez 2013).

The number and length of ARs in the ‘Mitchell’ cultivar are positively correlated with the initial total nitrogen content in the cuttings (Zerche et al. 2016). N deficiency reduces the levels of protein N in cuttings and the levels of amino acids, such as glutamine and asparagine, in fully developed leaves and the stem base. The corresponding inhibition of root meristematic cell differentiation into complete ARs supports the notion that reduced availability of amino acids in cuttings limits the synthesis of proteins needed for the differentiation and growth of ARs. Dark exposure of cuttings for 7 days before planting strongly enhances the number and length of ARs, based on the establishment of the first meristematic cells during the dark period and the subsequent accelerated differentiation and growth of ARs after planting and exposure to light (Klopotek et al. 2010). Irrespective of carbohydrate depletion during the dark period, dark‐incubated cuttings subsequently planted and cultivated under light allocate a higher proportion of dry matter to the roots than immediately planted cuttings (Klopotek et al. 2016). Under darkness, an increase in INVcw activity and the transcript accumulation of a specific INVcw isogene occur in the stem base, but not in the shoot apex, as a competing sink (Klopotek et al. 2016). Furthermore, high accumulation of amino acids such as asparagine and aspartate in fully developed leaves and the stem base as well as arginine in leaves is observed under darkness, which is associated with a decrease of N in insoluble protein in cuttings (Zerche et al. 2016). Based on these data, the authors developed a metabolic model of dark‐induced proteolysis. However, identification of the major control points of this process and the basis for its clear reversibility in petunia will require further investigation.

### Integrative model of the control of adventitious root formation in petunia

**Figure 2:**
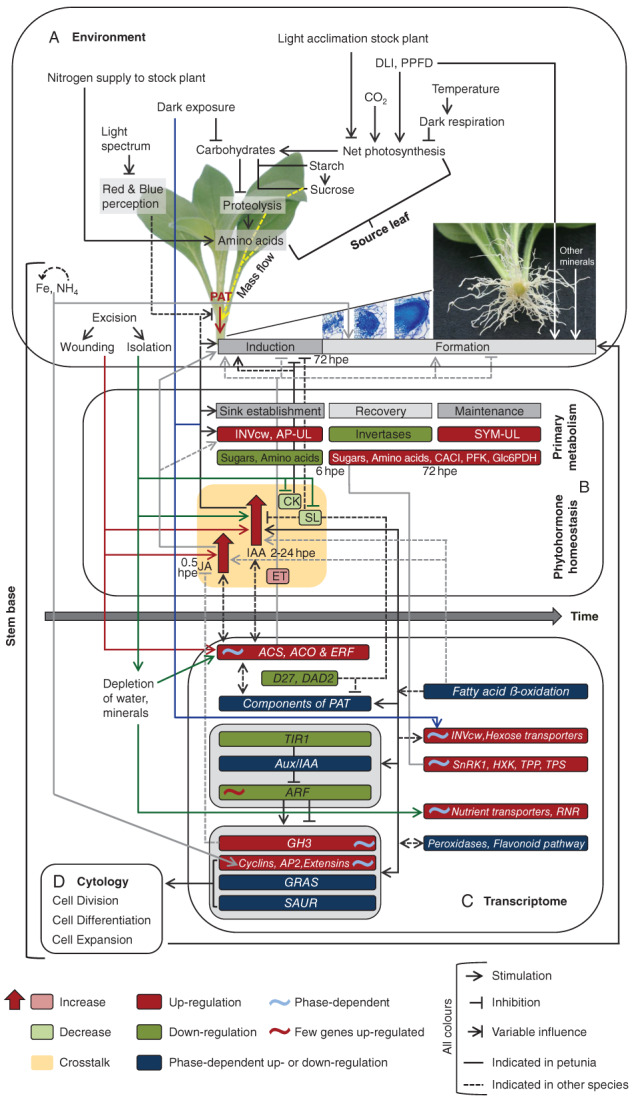
Current concept of the hormonal, metabolic and environmental regulation of AR formation in petunia cuttings, distinguishing between the levels of (A) environment, (B) primary metabolism and phytohormone homeostasis, (C) transcriptome and (D) cytology. Full lines in different colors indicate mechanistic relationships between environmental factors and diverse endogenous components, while arrowheads, crossbars or combination of both indicate stimulating, inhibitory or variable influences, respectively. Broken lines indicate hypothesized influences based on the literature on other plant species. Small pink and light green boxes indicate presumed increases and decreases in hormone levels, respectively, based on the literature on other plant species. Ochre shading indicates hormonal crosstalk. Rooting phases and the responses of plant metabolism and hormone homeostasis in the stem base are assigned to the time axis, while important time points under standard rooting conditions (Ahkami et al. 2009, 2013) are indicated as hours post excision (hpe). Red and green boxes indicate preferential up‐ vs down‐regulation of gene families or groups (in italic), metabolite levels or enzymatic activities. Dark blue boxes indicate gene families/groups whose up‐ or down‐regulation is generally phase dependent. Sinusoids indicate that specific genes within the box are upregulated (red color) or show phase dependency of upregulation (blue color). For gene or enzyme names, see the abbreviation list.

Based on the literature summarized above, we introduce a holistic concept of the control of AR formation in petunia cuttings (Fig. 2A–D) regarding the environmental (A), metabolic and hormonal (B), transcriptional (C) and cytological levels of regulation (D). Upon excision (Fig. 2A), wounding triggers the early accumulation of JA and ET in the stem base (Fig. 2B), the latter of which is supported by upregulation of genes coding for the ACC synthase (ACS) and for the ACC oxidase (ACO) (Fig. 2C). Based on the removal of the basipetal auxin drain, pre‐established PAT contributes to IAA accumulation (Fig. 2B), which is supported by early local de‐conjugation of IAA via upregulation of genes coding for IAA amino acid hydrolase (Fig. 2C).

Cutting isolation further reduces the levels of root‐derived CK and SL (Fig. 2B), and the level and perception of SL are further reduced via local down‐regulation of *D27* (encoding carotene isomerase D27) and *DAD2* (encoding decreased apical dominance 2) (Fig. 2C). During early induction, available CK and accumulating ET, acting via ethylene‐responsive transcription factors (ERF, Fig. 2C), may contribute to the dedifferentiation of AR competent cells (Fig. 2A). Thereafter, the rise of IAA in relation to the decreases in CK and SL causes induction of ARs and contributes to sink establishment in the stem base via activation of INVcw (Fig. 2A, B). Early JA accumulation (Fig. 2B) supports AR induction and may be involved in sink establishment. Intensive crosstalk between JA, auxin and ET (Fig. 2B) can be expected (Hoffmann et al. 2011, Gutierrez et al. 2012, Muday et al. 2012) while ET may exert an inhibitory influence during late induction (Fig. 2A, B; da Costa et al. 2013). Reduced water and mineral uptake and high auxin levels during induction are considered to stimulate prolonged upregulation of genes controlling ET biosynthesis and signaling (Fig. 2C), which may contribute to the differentiation of ARs while exerting an inhibitory effect on AR elongation (Fig. 2A).

Initial auxin accumulation initiates self‐regulatory adjustment of PAT components and important players in the auxin response machinery (Fig. 2C). While down‐regulation of genes coding for TIR1 and many ARFs results in desensitization against ‘auxin flush by accident’ (excision), the responses of specific genes coding for ARFs and Aux/IAA proteins control the reprogramming of specific cells towards AR formation as a survival strategy. Upregulation of *GH3* genes (Fig. 2C) contributes to the reduction of initially high IAA and JA levels through conversion to physiologically inactive amino acid conjugates (Staswick et al. 2005, Gutierrez et al. 2012), thereby providing the hormonal conditions for subsequent AR initiation and differentiation. Upregulation of peroxidase genes and changes in the expression of genes controlling the flavonoid pathway (Fig. 2C) further contribute to the reduction of IAA and modulation of PAT. Phase‐specific regulation of genes coding for cyclins, AP2 and GRAS transcription factors, extensins and SAUR proteins (Fig. 2C) mediates auxin‐controlled cell division, differentiation and expansion (Fig. 2D). Upregulation of *RIBONUCLEOTIDE REDUCTASE* genes (*RNR*, Fig. 2C) contributes to the production of deoxyribonucleotides needed for DNA synthesis.

Early induction of INVcw marks the establishment of the new sink characterized by AP‐UL (Fig. 2B), which, together with the corresponding upregulation of hexose transporter genes (Fig. 2C), channels locally available sugars and amino acids towards downstream metabolic pathways, while the upregulation of genes coding for hexokinase (HXK), sucrose non‐fermenting 1‐related protein kinase 1 (SnRK1), trehalose‐6‐phosphate phosphatase (TPP) and trehalose‐6‐phosphate synthase (TPS) indicates sugar signaling (Fig. 2C). During the recovery phase, the enhanced influx of sucrose powered by P_n_, and co‐transport (Fig. 2A) lead to the replenishment of sugars and amino acids, further supporting protein biosynthesis, while decreased INVcw activity indicates activation of SYM‐UL (Fig. 2B). The latter process is maintained during the maintenance phase, when high activities of phosphofructokinase (PFK) and glucose‐6‐phosphate dehydrogenase (GLc6PDH) and high levels of citrate cycle intermediates (CACI) reflect intensive carbon utilization in glycolysis, the pentose phosphate pathway and the citrate cycle. During the recovery and maintenance phases, the upregulation of genes encoding mineral transporters (Fig. 2C) supports nutrient acquisition. Mobilization of storage lipids via upregulation of genes controlling fatty acid ß oxidation (Fig. 2C) may further contribute to the early increases in JA and IAA (Ahkami et al. 2014).

At the whole‐cutting level, carbohydrate levels at the time of excision and P_n_ (the latter of which responds to the current PPFD, DLI, CO_2_ conditions and temperature‐mediated respiration and previous light acclimation) determine the level of sucrose in source leaves (which can be transported to the stem base), while the resulting mass flow in the phloem further drives amino acid transport (Fig. 2A). Light spectrum during the cultivation of cuttings affects source/sink relationships and AR formation, possibly via auxin homeostasis and signaling. Dark exposure of cuttings causes depletion of carbohydrates, which triggers low energy‐mediated proteolysis (Zerche et al. 2016), releasing free amino acids, particularly in the leaves of cuttings (Fig. 2A). At the same time, local upregulation of INVcw in the stem base enhances its sink competitiveness. The released amino acids are transported to the new sink, where they may promote AR formation after the dark period if proteolysis is stopped and carbohydrate depletion is rebalanced via photosynthesis. Increasing the N supply to stock plants (Fig. 2A) enhances the amount of amino acids that can be transported to the stem base. The local supply of Fe and NH_4_ stimulates early cell division involving upregulation of cyclins. The positive effects of combined fertilizer on AR development may involve rooting phase‐specific effects of those and also other individual nutrients.

## Research on arbuscular mycorrhiza in petunia

**Figure 3:**
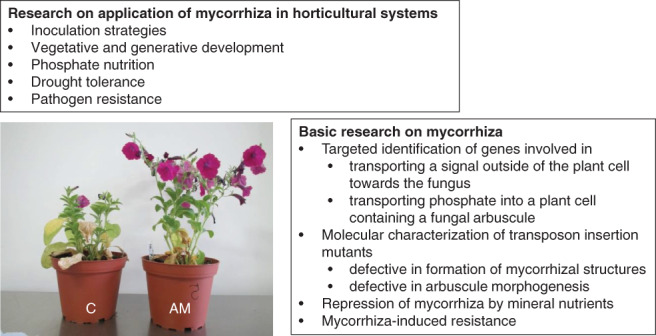
Mycorrhizal research with petunia as model. Photo shows two *Petunia inflata* plants inoculated (AM) or not (C) with an arbuscular mycorrhizal fungus. Positive growth response of the plant can be due to support of plant nutrition and/or due to increased tolerance and resistance to abiotic and biotic stress. Applied and basic research topics are listed and explained in the text.

Fossil findings and molecular data indicate that the first land plants were already colonized by AM fungi (Redecker et al. 2000). At present, 80% of vascular plant species form AM symbioses, and this type of mutualistic interaction can be found in all terrestrial ecosystems (Smith and Read 2008). AM fungi support nutrient uptake by plants, increase their resistance and tolerance to biotic and abiotic stresses and often show a positive impact on the nutritional value of plant products. Therefore, they are considered an important element of future sustainable plant production (Gianinazzi et al. 2010). Like all genera of the *Solanaceae* plant family, *Petunia* species form mutualistic symbioses with AM fungi and have been used to investigate basic, as well as applied research questions (Fig. 3).

*Petunia*‐AM symbioses were first noted in a study on the spread of viruses in different plants showing that virus titers were increased in mycorrhizal petunias compared with those in non‐mycorrhizal controls (Daft and Okusanya 1973). These authors did however, not mention effects on biomass or nutrient uptake. It was not until 1999 that mycorrhization of petunia plants appeared in the literature, but only inoculation strategies to achieve optimal colonization levels were tested in that study (Koide et al. 1999). In a study on the effect of mycorrhizal inoculation on different ornamental plant species, a decrease in flower numbers was recorded but was accompanied by a colonization level of up to 85% (Gaur and Adholeya 2005). This phenomenon was not observed again in follow‐up studies on the use of mycorrhiza in petunia production systems. Shamshiri et al. (2011) showed a general positive impact of mycorrhization on vegetative and generative development and an increase in drought tolerance. Using a soilless culture system, as usually applied in horticultural practice, it was found that the amount of phosphate in fertilizer could be reduced by half while still achieving the same biomass and phosphate content of petunia plants if the plants were inoculated with an AM fungus (Hayek et al. 2012).

It has been demonstrated that petunia is a suitable model for research on plant development and plant functions (Gerats and Vandenbussche 2005). Petunia has also been employed for studying mechanisms underlying mycorrhizal interactions. This research focused on three aspects of these interactions: the formation of AM symbiosis, the support of plant nutrition by AM fungi and mycorrhiza‐induced resistance (MIR).

The first step of the mycorrhizal interaction is the reception of SL in root exudates by the AM fungus, which enables hyphal branching and, consequently, physical contact with the roots (Akiyama et al. 2005). Kretzschmar et al. (2012) identified the ABC transporter PDR1 in petunia, which transports SL out of the cell. This protein is highly abundant in roots under phosphate‐deficient conditions, and the *pdr1* mutant shows reduced mycorrhizal colonization. After physical contact, the fungus penetrates root epidermal cells, and hyphae spread inside the root cortex and finally form tree‐like structures in the apoplast of the inner root cortical cells (Genre et al. 2008, Fig. 1E). These so‐called arbuscules are important sites for nutrient exchange between the symbiotic partners (Smith and Read 2008). Screening of a population of transposon insertion mutants for defects in root colonization by AM fungi resulted in the identification of the *pam1* mutant (Reddy et al. 2007). *pam1* was among the first mycorrhiza mutants not identified in a leguminous plant, based on the fact that nodule phenotypes are often accompanied by mycorrhizal phenotypes. This mutant shows reduced penetration of the root epidermis by the fungus and absence of any arbuscules. Cloning of the corresponding gene showed that *PAM1* encodes a VAPYRIN homologue that localizes to spherical structures associated with the tonoplast (Feddermann et al. 2010). PAM1 is thought to play a role in transporting components required for the penetration of the fungus into the apoplast during colonization and arbuscule formation. Further screening of the transposon insertion population for mycorrhizal phenotypes resulted in the identification of a mutant that is defective in arbuscule morphogenesis and does not prevent additional colonization of meristems by the AM fungus (Rich et al. 2015). The corresponding *ATA/RAM1* gene encodes a GRAS transcription factor that regulates numerous genes involved in mycorrhizal development and functions and therefore appears to be critical for mycorrhiza formation (Rich et al. 2017).

AM symbioses are characterized by the exchange of nutrients, in which the fungus mainly provides phosphate to the plant (Marschner and Dell 1994). The identification of mycorrhiza‐regulated phosphate transporters in plants is therefore a central task in mycorrhizal research. Using a PCR‐cloning approach with degenerate primers, five corresponding genes were cloned (Wegmueller et al. 2008). Two of these genes were induced by mycorrhization, while one was mycorrhiza‐specific. This situation corresponds to findings in tomato and potato (Nagy et al. 2005) but is different from that in plants of other families, such as barrel medic (Harrison et al. 2002) and rice (Paszkowski et al. 2002), in which only one mycorrhiza‐specific phosphate transporter gene exists.

It has been shown in many plants that fertilization with a sufficient amount of phosphate for the plant represses mycorrhizal colonization; this is also true for petunia (Koide et al. 1999). To elucidate the mechanisms underlying the inhibition of the spreading of the fungus within roots, mycorrhizal petunia plants were fertilized with high amounts of phosphate, and the response concerning colonization patterns and gene expression was investigated using an array carrying nearly 25 000 unigene sequences (Breuillin et al. 2010). The results revealed that phosphate fertilization led to the formation of truncated arbuscules after 1 week, which was preceded by the repression of genes involved in symbiotic functions, encoding, for example, mycorrhiza‐regulated phosphate transporters and enzymes involved in the biosynthesis of SL. Further analyses of the interplay between mycorrhizal symbiosis and phosphate nutrition were targeted to the involvement of lysophosphatidylcholine as a regulator (Tan et al. 2012) and of microRNAs (Gu et al. 2014). AM fungi not only transport phosphate towards the plant but also supply a number of other mineral elements (Marschner and Dell 1994). Nouri et al. (2014) showed that in petunia plants in which mycorrhizal colonization was repressed by phosphate fertilization, this repression was at least partially released by other nutrient deficiencies. This effect was most prominent for nitrogen and was also significant for potassium, calcium and iron.

Mycorrhiza‐induced resistance (MIR) against root pathogens represents an important feature of these symbioses and is one reason to apply AM fungi in plant production systems. MIR is important because root pathogens and parasites are difficult to combat. The application of pesticides is critical or even forbidden, and very few resistant cultivars exist. Different pathosystems have been used to study MIR, and it is intended to also use the model plant petunia for this purpose. *Thielaviopsis basicola* is a pathogenic fungus of petunia that was identified quite some time ago (Johnson 1916) but is still difficult to control (Hsi and Ortiz 1980). Hayek et al. (2012) demonstrated MIR against *T. basicola* in petunia roots. A significant reduction of disease symptoms (85%) was observed under these conditions, but interestingly, only for one out of three AM fungal isolates. Hence, petunia is an ideal system for studying MIR because general mycorrhiza‐regulated responses can be distinguished from responses specifically associated with MIR in these plants. The first gene expression studies using this system pointed to particular members of gene families encoding chitinases and lipoxygenases that could play a role in MIR (Hayek et al. 2014).

## Conclusions

During the past decade, the utilization of *P. hybrida* as a model system has led to discoveries that have substantially contributed to the understanding of AR formation in shoot tip cuttings as well as the regulation and function of AM symbiosis. Current knowledge and concepts, together with the newly available petunia genome sequence and the constantly growing functional genomics platform, provide an excellent outlook for the full exploitation of the petunia system in this context.

Based on current concepts, the petunia system not only allows intense focus on specific processes at the cellular level but also supports holistic analyses of root initiation, development and function in the highly controlled environment provided by modern horticulture. Thus, mechanistic models of processes and environmental responses can be further elaborated. For this purpose, it will be important in the future to use all components available in the toolbox. On the one hand, screening and analysis of mutants for research on AM symbioses can be applied to decipher the molecular‐genetic background of AR formation. On the other hand, sophisticated physiological analyses established for cuttings could be used to understand the phytohormonal regulation and metabolic processes involved in mycorrhizal symbioses. The screening of RILs and GWAS of cultivars for the identification of candidate genes can be applied in both fields of research. The role of putative candidate genes can be rapidly tested by analyzing transposon insertion mutants. Subsequently, the function of these genes and their gene products can be investigated in detail by applying a wide range of transgenic approaches established for petunia, including the stable and transient expression of promoter‐reporter constructs and protein‐GFP fusions as well as site‐directed mutagenesis.

One important issue in mycorrhizal research that can be now approached is the molecular basis of particular responses to inoculation by AM fungi. It has been observed that *P. axillaris* and *P. inflata* show contrasting growth responses and quantitative differences in mycorrhiza‐supported P‐uptake and mycorrhiza‐induced resistance against a root pathogen (Camehl and Franken, unpublished). Interestingly, the same species show different intensities of AR formation and dark responses (Druege, unpublished). Therefore, it may be possible to identify genes and to study the molecular processes underlying these phenomena. Such research will further deepen our insight into AR formation and mycorrhizal symbiosis and extend the current models related to these two important topics in plant sciences. The combination of genetic and environmental approaches may advance both the breeding of new cultivars with improved responses of root development and function to specific abiotic and biotic factors and the development of more sophisticated technologies to optimize horticultural production chains, thus increasing ecological and economic sustainability in the production and processing of petunia and of other important crops.

## Author contributions

U.D. developed the concept of the review, designed Figs. 1 and 2, wrote the chapter related to adventitious rooting and contributed to the other chapters. P.F. designed Fig. 3., wrote the chapter related to mycorrhiza and contributed to the other chapters.

## References

[ppl12762-bib-0001] Ahkami AH , Lischewski S , Haensch K‐T , Porfirova S , Hofmann J , Rolletschek H , Melzer M , Franken P , Hause B , Druege U , Hajirezaei MR (2009) Molecular physiology of adventitious root formation in *Petunia hybrida* cuttings: involvement of wound response and primary metabolism. New Phytol 181: 613–625 19076299 10.1111/j.1469-8137.2008.02704.x

[ppl12762-bib-0002] Ahkami AH , Melzer M , Ghaffari MR , Pollmann S , Javid MG , Shahinnia F , Hajirezaei MR , Druege U (2013) Distribution of indole‐3‐acetic acid in *Petunia hybrida* shoot tip cuttings and relationship between auxin transport, carbohydrate metabolism and adventitious root formation. Planta 238: 499–517 23765266 10.1007/s00425-013-1907-z PMC3751230

[ppl12762-bib-0003] Ahkami AH , Scholz U , Steuernagel B , Strickert M , Haensch K‐T , Druege U , Reinhardt D , Nouri E , von Wirén N , Franken P , Hajirezaei MR (2014) Comprehensive transcriptome analysis unravels the existence of crucial genes regulating primary metabolism during adventitious root formation in *Petunia hybrida* . PLoS One 9: e100997 24978694 10.1371/journal.pone.0100997 PMC4076263

[ppl12762-bib-0004] Akiyama K , Matsuzaki K , Hayashi H (2005) Plant sesquiterpenes induce hyphal branching in arbuscular mycorrhizal fungi. Nature 435: 824–827 15944706 10.1038/nature03608

[ppl12762-bib-0005] Al‐Babili S , Bouwmeester HJ (2015) Strigolactones, a novel carotenoid‐derived plant hormone. Annu Rev Plant Biol 66: 161–186 25621512 10.1146/annurev-arplant-043014-114759

[ppl12762-bib-0006] Bashandy H , Teeri TH (2017) Genetically engineered orange petunias on the market. Planta 246: 277–280 28647812 10.1007/s00425-017-2722-8 PMC5522655

[ppl12762-bib-0007] BMEL (2014) Horticulture in Germany – Facts and Figures. Report Federal Ministry of Food and Agriculture. Available at https://www.bmel.de/SharedDocs/Downloads/EN/Publications/HorticultureGermany‐Brochure.pdf;jsessionid=54D83BA51870990DF914905242AAB784.2_cid358?__blob=publicationFile (accessed 3 May 2018)

[ppl12762-bib-0008] Bombarely A , Moser M , Amrad A , Bao M , Bapaume L , Barry CS , Bliek M , Boersma MR , Borghi L , Bruggmann R , Bucher M , D'Agostino N , Davies K , Druege U , Dudareva N , Egea‐Cortines M , Delledonne M , Fernandez‐Pozo N , Franken P , Grandont L , Heslop‐Harrison JS , Hintzsche J , Johns M , Koes R , Lv X , Lyons E , Malla D , Martinoia E , Mattson NS , Morel P , Mueller LA , Muhlemann J , Nouri E , Passeri V , Pezzotti M , Qi Q , Reinhardt D , Rich M , Richert‐Pöggeler KR , Robbins TP , Schatz MC , Schranz ME , Schuurink RC , Schwarzacher T , Spelt K , Tang H , Urbanus SL , Vandenbussche M , Vijverberg K , Villarino GH , Warner RM , Weiss J , Yue Z , Zethof J , Quattrocchio F , Sims TL , Kuhlemeier C (2016) Insight into the evolution of the *Solanaceae* from the parental genomes of *Petunia hybrida* . Nat Plants 2: 16074 10.1038/nplants.2016.74 27255838

[ppl12762-bib-0009] Breuillin F , Schramm J , Hajirezaei M , Ahkami A , Favre P , Druege U , Hause B , Bucher M , Kretzschmar T , Bossolini E , Kuhlemeier C , Martinoia E , Franken P , Scholz U , Reinhardt D (2010) Phosphate systemically inhibits development of arbuscular mycorrhiza in *Petunia hybrida* and represses genes involved in mycorrhizal functioning. Plant J 64: 1002–1017 21143680 10.1111/j.1365-313X.2010.04385.x

[ppl12762-bib-0010] Clark DG , Gubrium EK , Barrett JE , Nell TA , Klee HJ (1999) Root formation in ethylene‐insensitive plants. Plant Physiol 121: 53–60 10482660 10.1104/pp.121.1.53 PMC59389

[ppl12762-bib-0011] Clark DG , Dervinis C , Barret JE , Klee H , Jones M (2004) Drought‐induced leaf senescence and horticultural performance of transgenic P‐SAG12‐IPT petunias. J Am Soc Hortic Sci 129: 93–99

[ppl12762-bib-0012] Correa LD , Troleis J , Mastroberti AA , Mariath JEA , Fett AG (2012) Distinct modes of adventitious rooting in *Arabidopsis thaliana* . Plant Biol 14: 100–109 21974782 10.1111/j.1438-8677.2011.00468.x

[ppl12762-bib-0013] da Costa CT , de Almeida MR , Ruedell CM , Schwambach J , Maraschin FS , Fett‐Neto AG (2013) When stress and development go hand in hand: main hormonal controls of adventitious rooting in cuttings. Front Plant Sci 4: 133 23717317 10.3389/fpls.2013.00133 PMC3653114

[ppl12762-bib-0014] Currey CJ , Lopez RG (2013) Cuttings of *Impatiens, Pelargonium*, and *Petunia* propagated under light‐emitting diodes and high‐pressure sodium lamps have comparable growth, morphology, gas exchange, and post‐transplant performance. HortScience 48: 428–434

[ppl12762-bib-0015] Currey CJ , Lopez RG (2015) Biomass accumulation and allocation, photosynthesis, and carbohydrate status of new Guinea impatiens, geranium, and petunia cuttings are affected by photosynthetic daily light integral during root development. J Am Soc Hort Sci 140: 542–549

[ppl12762-bib-0016] Daft MJ , Okusanya BO (1973) Effect of endogone mycorrhiza on plant growth. V. Influence of infection on the multiplication of viruses in tomato, petunia and strawberry. New Phytol 72: 975–983

[ppl12762-bib-0017] Della Rovere F , Fattorini L , D'Angeli S , Veloccia A , Falasca G , Altamura MM (2013) Auxin and cytokinin control formation of the quiescent Centre in the adventitious root apex of *Arabidopsis* . Ann Bot 112: 1395–1407 24061489 10.1093/aob/mct215 PMC3806543

[ppl12762-bib-0018] Dimasi‐Theriou K , Economou AS , Sfakiotakis EM (1993) Promotion of petunia (*Petunia hybrida* L.) regeneration in vitro by ethylene. Plant Cell, Tissue Organ Cult 32: 219–225

[ppl12762-bib-0019] Druege U , Franken P , Lischewski S , Ahkami AH , Zerche S , Hause B , Hajirezaei MR (2014) Transcriptomic analysis reveals ethylene as stimulator and auxin as regulator of adventitious root formation in petunia cuttings. Front Plant Sci 5: 494 25400641 10.3389/fpls.2014.00494 PMC4212214

[ppl12762-bib-0020] Feddermann N , Muni RRD , Zeier T , Stuurman J , Ercolin F , Schorderet M , Reinhardt D (2010) The PAM1 gene of petunia, required for intracellular accommodation and morphogenesis of arbuscular mycorrhizal fungi, encodes a homologue of VAPYRIN. Plant J 64: 470–481 20804456 10.1111/j.1365-313X.2010.04341.x

[ppl12762-bib-0021] Galliot C , Stuurman J , Kuhlemeier C (2006) The genetic dissection of floral pollination syndromes. Curr Opin Plant Biol 9: 78–82 16325455 10.1016/j.pbi.2005.11.003

[ppl12762-bib-0022] Gaur A , Adholeya A (2005) Diverse response of five ornamental plant species to mixed indigenous and single isolate arbuscular‐mycorrhizal inocula in marginal soil amended with organic matter. J Plant Nutr 28: 707–723

[ppl12762-bib-0023] Genre A , Chabaud M , Faccio A , Barker DG , Bonfante P (2008) Prepenetration apparatus assembly precedes and predicts the colonization patterns of arbuscular mycorrhizal fungi within the root cortex of both *Medicago truncatula* and *Daucus carota* . Plant Cell 20: 1407–1420 18515499 10.1105/tpc.108.059014 PMC2438458

[ppl12762-bib-0024] Gerats T , Vandenbussche M (2005) A model system comparative for research: *Petunia* . Trends Plant Sci 10: 251–256 15882658 10.1016/j.tplants.2005.03.005

[ppl12762-bib-0025] Gerats AGM , Huits H , Vrijlandt E , Marana C , Souer E , Beld M (1990) Molecular characterization of a nonautonomous transposable element (dTPH1) of petunia. Plant Cell 2: 1121–1128 1967052 10.1105/tpc.2.11.1121 PMC159959

[ppl12762-bib-0026] Gerats T , Zethof J , Vandenbussche M (2013) Identification and applications of the petunia class II Act1/dTph1 transposable element system In: Peterson T (ed) Plant Transposable Elements: Methods and Protocols, Methods in Molecular Biology. Springer Science+Business Media, New York, pp 223–237 10.1007/978-1-62703-568-2_16 23918432

[ppl12762-bib-0027] Gianinazzi S , Gollotte A , Binet MN , van Tuinen D , Redecker D , Wipf D (2010) Agroecology: the key role of arbuscular mycorrhizas in ecosystem services. Mycorrhiza 20: 519–530 20697748 10.1007/s00572-010-0333-3

[ppl12762-bib-0029] Gu M , Liu W , Meng Q , Zhang W , Chen A , Sun S , Xu G (2014) Identification of microRNAs in six solanaceous plants and their potential link with phosphate and mycorrhizal signaling. J Integr Plant Biol 56: 1164–1178 24975554 10.1111/jipb.12233

[ppl12762-bib-0030] Guo YF , Lin WK , Chen QX , Vallejo VA , Warner RM (2017) Genetic determinants of crop timing and quality traits in two interspecific *Petunia* recombinant inbred line populations. Sci Rep 7: 3200 28600539 10.1038/s41598-017-03528-9 PMC5466624

[ppl12762-bib-0031] Gutierrez L , Mongelard G , Flokova K , Pacurar DI , Novak O , Staswick P , Kowalczyk M , Pacurar M , Demailly H , Geiss G , Bellini C (2012) Auxin controls *Arabidopsis* adventitious root initiation by regulating jasmonic acid homeostasis. Plant Cell 24: 2515–2527 22730403 10.1105/tpc.112.099119 PMC3406919

[ppl12762-bib-0032] Harrison MJ , Dewbre GR , Liu JY (2002) A phosphate transporter from *Medicago truncatula* involved in the acquisiton of phosphate released by arbuscular mycorrhizal fungi. Plant Cell 14: 2413–2429 12368495 10.1105/tpc.004861 PMC151226

[ppl12762-bib-0033] Hayek S , Grosch R , Gianinazzi‐Pearson V , Franken P (2012) Bioprotection and alternative fertilisation of petunia using mycorrhiza in a soilless production system. Agron Sustain Dev 32: 765–771

[ppl12762-bib-0034] Hayek S , Gianinazzi‐Pearson V , Gianinazzi S , Franken P (2014) Elucidating mechanisms of mycorrhiza‐induced resistance against *Thielaviopsis basicola* via targeted transcript analysis of *Petunia hybrida* genes. Physiol Mol Plant Pathol 88: 67–76

[ppl12762-bib-0035] Hilo A , Shahinnia F , Druege U , Franken P , Melzer M , Rutten T , von Wiren N , Hajirezaei MR (2017) A specific role of iron in promoting meristematic cell division during adventitious root formation. J Exp Bot 68: 4233–4247 28922771 10.1093/jxb/erx248 PMC5853222

[ppl12762-bib-0036] Hoffmann M , Hentrich M , Pollmann S (2011) Auxin‐oxylipin crosstalk: relationship of antagonists. J Integr Plant Biol 53: 429–445 21658177 10.1111/j.1744-7909.2011.01053.x

[ppl12762-bib-0037] Horsch RB , Fry JE , Hoffmann NL , Eichholtz D , Rogers SG , Fraley RT (1985) A simple and general method for transferring genes into plants. Science 227: 1229–1231 17757866 10.1126/science.227.4691.1229

[ppl12762-bib-0038] Hsi DCH , Ortiz MJ (1980) Suppression of *Thielaviopsis basicola* by 2 fungizides to sandy loam soils in New Mexico USA. Plant Dis 64: 1011–1012

[ppl12762-bib-0039] Jasanoff S (2005) Designs on Nature. Princeton University Press, Princeton

[ppl12762-bib-0040] Johnson J (1916) Host plants of *Thielavia basicola* . J Agric Res 7: 289–300

[ppl12762-bib-0042] Jussieu AL (1803) Sur le petunia, genre nouveau de la famille des plantes solanees. Ann Mus Natl Hist Nat 2: 214–216

[ppl12762-bib-0043] Kaul S , Koo HL , Jenkins J , Rizzo M , Rooney T , Tallon LJ , Feldblyum T , Nierman W , Benito MI , Lin XY , Town CD , Venter JC , Fraser CM , Tabata S , Nakamura Y , Kaneko T , Sato S , Asamizu E , Kato T , Kotani H , Sasamoto S , Ecker JR , Theologis A , Federspiel NA , Palm CJ , Osborne BI , Shinn P , Conway AB , Vysotskaia VS , Dewar K , Conn L , Lenz CA , Kim CJ , Hansen NF , Liu SX , Buehler E , Altafi H , Sakano H , Dunn P , Lam B , Pham PK , Chao Q , Nguyen M , Yu GX , Chen HM , Southwick A , Lee JM , Miranda M , Toriumi MJ , Davis RW , Wambutt R , Murphy G , Dusterhoft A , Stiekema W , Pohl T , Entian KD , Terryn N , Volckaert G , Salanoubat M , Choisne N , Rieger M , Ansorge W , Unseld M , Fartmann B , Valle G , Artiguenave F , Weissenbach J , Quetier F , Wilson RK , de la Bastide M , Sekhon M , Huang E , Spiegel L , Gnoj L , Pepin K , Murray J , Johnson D , Habermann K , Dedhia N , Parnell L , Preston R , Hillier L , Chen E , Marra M , Martienssen R , McCombie WR , Mayer K , White O , Bevan M , Lemcke K , Creasy TH , Bielke C , Haas B , Haase D , Maiti R , Rudd S , Peterson J , Schoof H , Frishman D , Morgenstern B , Zaccaria P , Ermolaeva M , Pertea M , Quackenbush J , Volfovsky N , Wu DY , Lowe TM , Salzberg SL , Mewes HW , Rounsley S , Bush D , Subramaniam S , Levin I , Norris S , Schmidt R , Acarkan A , Bancroft I , Quetier F , Brennicke A , Eisen JA , Bureau T , Legault BA , Le QH , Agrawal N , Yu Z , Martienssen R , Copenhaver GP , Luo S , Pikaard CS , Preuss D , Paulsen IT , Sussman M , Britt AB , Selinger DA , Pandey R , Mount DW , Chandler VL , Jorgensen RA , Pikaard C , Juergens G , Meyerowitz EM , Theologis A , Dangl J , Jones JDG , Chen M , Chory J , Somerville MC , Ar Gen I (2000) Analysis of the genome sequence of the flowering plant *Arabidopsis thaliana* . Nature 408: 796–815 11130711 10.1038/35048692

[ppl12762-bib-0044] Klopotek Y , Haensch K‐T , Hause B , Hajirezaei M‐R , Druege U (2010) Dark exposure of petunia cuttings strongly improves adventitious root formation and enhances carbohydrate availability during rooting in the light. J Plant Physiol 167: 547–554 20047776 10.1016/j.jplph.2009.11.008

[ppl12762-bib-0045] Klopotek Y , George E , Druege U , Klaering H‐P (2012) Carbon assimilation of petunia cuttings in a non‐disturbed rooting environment: response to environmental key factors and adventitious root formation. Sci Hortic 145: 118–126

[ppl12762-bib-0046] Klopotek Y , Franken P , Klaering H‐P , Fischer K , Hause B , Hajirezaei MR , Druege U (2016) A higher sink competitiveness of the rooting zone and invertases are involved in dark stimulation of adventitious root formation in *Petunia hybrida* cuttings. Plant Sci 243: 10–22 26795147 10.1016/j.plantsci.2015.11.001

[ppl12762-bib-0047] Kohlen W , Charnikhova T , Lammers M , Pollina T , Toth P , Haider I , Pozo MJ , de Maagd RA , Ruyter‐Spira C , Bouwmeester HJ , Lopez‐Raez JA (2012) The tomato CAROTENOID CLEAVAGE DIOXYGENASE8 (SlCCD8) regulates rhizosphere signaling, plant architecture and affects reproductive development through strigolactone biosynthesis. New Phytol 196: 535–547 22924438 10.1111/j.1469-8137.2012.04265.x

[ppl12762-bib-0048] Koide RT , Landherr LL , Besmer YL , Detweiler JM , Holcomb EJ (1999) Strategies for mycorrhizal inoculation of six annual bedding plant species. HortScience 34: 1217–1220

[ppl12762-bib-0049] Kretzschmar T , Kohlen W , Sasse J , Borghi L , Schlegel M , Bachelier JB , Reinhardt D , Bours R , Bouwmeester HJ , Martinoia E (2012) A petunia ABC protein controls strigolactone‐dependent symbiotic signaling and branching. Nature 483: 341–344 22398443 10.1038/nature10873

[ppl12762-bib-0050] Linn F , Heidmann I , Saedler H , Meyer P (1990) Epigenetic changes in the expression of the maize A1 gene in *Petunia hybrida* ‐ role of numbers of integrated gene copies and state of methylation. Mol Gen Genet 222: 329–336 1703268 10.1007/BF00633837

[ppl12762-bib-0051] Lischweski S , Muchow A , Guthörl D , Hause B (2015) Jasmonates act positively in adventitious root formation in petunia cuttings. BMC Plant Biol 15: 229 26394764 10.1186/s12870-015-0615-1 PMC4579608

[ppl12762-bib-0052] de Lucas M , Prat S (2014) PIFs get BRight: PHYTOCHROME INTERACTING FACTORs as integrators of light and hormonal signals. New Phytol 202: 1126–1141 24571056 10.1111/nph.12725

[ppl12762-bib-0053] Marschner P (2012) Marschner's Mineral Nutrition of Higher Plants, 3rd Edn. Academic Press, Boston

[ppl12762-bib-0054] Marschner H , Dell B (1994) Nutrient‐uptake in mycorrhizal symbiosis. Plant and Soil 159: 89–102

[ppl12762-bib-0055] Meyer P , Heidmann I , Forkmann G , Saedler H (1987) A new petunia flower color generated by transformation of a mutant with a maize gene. Nature 330: 677–678 3683587 10.1038/330677a0

[ppl12762-bib-0056] Muday GK , Rahman A , Binder BM (2012) Auxin and ethylene: collaborators or competitors? Trends Plant Sci 17: 181–195 22406007 10.1016/j.tplants.2012.02.001

[ppl12762-bib-0057] Nagy R , Karandashov V , Chague W , Kalinkevich K , Tamasloukht M , Xu GH , Jakobsen I , Levy AA , Amrhein N , Bucher M (2005) The characterization of novel mycorrhiza‐specific phosphate transporters from *Lycopersicon esculentum* and *Solanum tuberosum* uncovers functional redundancy in symbiotic phosphate transport in solanaceous species. Plant J 42: 236–250 15807785 10.1111/j.1365-313X.2005.02364.x

[ppl12762-bib-0059] Nouri E , Breuillin‐Sessoms F , Feller U , Reinhardt D (2014) Phosphorus and nitrogen regulate arbuscular mycorrhizal symbiosis in *Petunia hybrida* . PLoS One 9: e90841 24608923 10.1371/journal.pone.0090841 PMC3946601

[ppl12762-bib-0060] Paszkowski U , Kroken S , Roux C , Briggs SP (2002) Rice phosphate transporters include an evolutionarily divergent gene specifically activated in arbuscular mycorrhizal symbiosis. Proc Natl Acad Sci USA 99: 13324–13329 12271140 10.1073/pnas.202474599 PMC130632

[ppl12762-bib-0061] Rasmussen A , Mason MG , De Cuyper C , Brewer PB , Herold S , Agusti J , Geelen D , Greb T , Goormachtig S , Beeckman T , Beveridge CA (2012) Strigolactones suppress adventitious rooting in *Arabidopsis* and pea. Plant Physiol 158: 1976–1987 22323776 10.1104/pp.111.187104 PMC3320200

[ppl12762-bib-0062] Reddy DMRS , Schorderet M , Feller U , Reinhardt D (2007) A petunia mutant affected in intracellular accommodation and morphogenesis of arbuscular mycorrhizal fungi. Plant J 51: 739–750 17573800 10.1111/j.1365-313X.2007.03175.x

[ppl12762-bib-0063] Redecker D , Kodner R , Graham LE (2000) Glomalean fungi from the Ordovician. Science 289: 1920–1921 10988069 10.1126/science.289.5486.1920

[ppl12762-bib-0065] Rich MK , Schorderet M , Bapaume L , Falquet L , Morel P , Vandenbussche M , Reinhardt D (2015) The *Petunia* GRAS transcription factor ATA/RAM1 regulates symbiotic gene expression and fungal morphogenesis in arbuscular mycorrhiza. Plant Physiol 168: 788–797 25971550 10.1104/pp.15.00310 PMC4741351

[ppl12762-bib-0066] Rich MK , Courty PE , Roux C , Reinhardt D (2017) Role of the GRAS transcription factor ATA/RAM1 in the transcriptional reprogramming of arbuscular mycorrhiza in *Petunia hybrida* . BMC Genom 18: 589 10.1186/s12864-017-3988-8 PMC5549340 28789611

[ppl12762-bib-0067] Santos KM , Fisher PR , Argo WR (2009) Stem versus foliar uptake during propagation of *Petunia* x *hybrida* vegetative cuttings. Hortscience 44: 1974–1977

[ppl12762-bib-0068] Segatto ALA , Ramos‐Fregonezi AMC , Bonatto SL , Freitas LB (2014) Molecular insights into the purple‐flowered ancestor of garden petunias. Am J Bot 101: 119–127 24368755 10.3732/ajb.1300223

[ppl12762-bib-0069] Shamshiri MH , Mozafari V , Sedaghati E , Bagheri V (2011) Response of petunia plants (*Petunia hybrida* cv. Mix) inoculated with *Glomus mosseae* and *Glomus intraradices* to phosphorous and drought stress. J Agric Sci Tech 13: 929–942

[ppl12762-bib-0070] Shibuya K , Barry KG , Ciardi JA , Loucas HM , Underwood BA , Nourizadeh S , Ecker JR , Klee HJ , Clark DG (2004) The central role of PhEIN2 in ethylene responses throughout plant development in petunia. Plant Physiol 136: 2900–2912 15466231 10.1104/pp.104.046979 PMC523352

[ppl12762-bib-0071] Smith S , Read D (2008) Mycorrhizal Symbiosis, 3rd Edn. Academic Press, New York

[ppl12762-bib-0072] Spitzer B , Zvi MMB , Ovadis M , Marhevka E , Barkai O , Edelbaum O , Marton I , Masci T , Alon M , Morin S , Rogachev I , Aharoni A , Vainstein A (2007) Reverse genetics of floral scent: application of tobacco rattle virus‐based gene silencing in petunia. Plant Physiol 145: 1241–1250 17720754 10.1104/pp.107.105916 PMC2151718

[ppl12762-bib-0073] Staswick PE , Serban B , Rowe M , Tiryaki I , Maldonado MT , Maldonado MC , Suza W (2005) Characterization of an *Arabidopsis* enzyme family that conjugates amino acids to indole‐3‐acetic acid. Plant Cell 17: 616–627 15659623 10.1105/tpc.104.026690 PMC548830

[ppl12762-bib-0074] Steffens B , Rasmussen A (2016) The physiology of adventitious roots. Plant Physiol 170: 603–617 26697895 10.1104/pp.15.01360 PMC4734560

[ppl12762-bib-0075] Tan Z , Hu Y , Lin Z (2012) PhPT4 is a mycorrhizal‐phosphate transporter suppressed by lysophosphatidylcholine in petunia roots. Plant Mol Biol Rep 30: 1480–1487

[ppl12762-bib-0076] Vallejo VA , Tychonievich J , Lin WK , Wangchu L , Barry CS , Warner RM (2015) Identification of QTL for crop timing and quality traits in an interspecific *Petunia* population. Mol Breed 35: 2

[ppl12762-bib-0077] Vandenbussche M , Janssen A , Zethof J , van Orsouw N , Peters J , van Eijk MJT , Rijpkema AS , Schneiders H , Santhanam P , de Been M , van Tunen A , Gerats T (2008) Generation of a 3D indexed *Petunia* insertion database for reverse genetics. Plant J 54: 1105–1114 18346192 10.1111/j.1365-313X.2008.03482.x

[ppl12762-bib-0078] Vandenbussche M , Chambrier P , Bento SR , Morel P (2016) Petunia, your next supermodel? Front Plant Sci 7: 72 26870078 10.3389/fpls.2016.00072 PMC4735711

[ppl12762-bib-0079] Verstraeten I , Beeckman T , Geelen D (2013) Adventitious root induction in *Arabidopsis thaliana* as a model for in vitro root organogenesis In: De Smet I (ed) Plant Organogenesis: Methods and Protocols, Methods in Molecular Biology, Vol. 959 Springer Science+Business Media, New York, pp 159–175 10.1007/978-1-62703-221-6_10 23299674

[ppl12762-bib-0080] Verweij W , Di Sansebastiano GP , Quattrocchio F , Dalessandro G (2008) *Agrobacterium*‐mediated transient expression of vacuolar GFPs in *Petunia* leaves and petals. Plant Biosyst 142: 343–347

[ppl12762-bib-0081] Warner RM , Walworth AE (2010) Quantitative inheritance of crop timing traits in interspecific hybrid *Petunia* populations and interactions with crop quality parameters. J Hered 101: 308–316 20142456 10.1093/jhered/esp131

[ppl12762-bib-0082] Wegmueller S , Svistoonoff S , Reinhardt D , Stuurman J , Amrhein N , Bucher M (2008) A transgenic dTph1 insertional mutagenesis system for forward genetics in mycorrhizal phosphate transport of *Petunia* . Plant J 54: 1115–1127 18315538 10.1111/j.1365-313X.2008.03474.x

[ppl12762-bib-0084] Zerche S , Haensch K‐T , Druege U , Hajirezaei MR (2016) Nitrogen remobilisation facilitates adventitious root formation on reversible dark‐induced carbohydrate depletion in *Petunia hybrida* . BMC Plant Biol 16: 219 27724871 10.1186/s12870-016-0901-6 PMC5056478

[ppl12762-bib-0085] Zhang B , Yang X , Yang CP , Li MY , Guo YL (2016) Exploiting the CRISPR/Cas9 system for targeted genome mutagenesis in *Petunia* . Sci Rep 6: 20315 10.1038/srep20315 PMC4738242 26837606

